# Knowledge, attitude, and prevention practices of cutaneous leishmaniasis in highly-endemic rural areas of Kandahar province, Afghanistan: A large cross-sectional community-based study

**DOI:** 10.1371/journal.pntd.0013575

**Published:** 2026-06-03

**Authors:** Bilal Ahmad Rahimi, Sharafuddin Resha, Mohammadullah Khaksar, Khwaja Mir Islam Saeed, Hashmatullah Osmani, Abdul Baqi Daqiq, Habiburahman Rahmani, Hikmatullah Saleem, Walter R. Taylor

**Affiliations:** 1 Faculty of Medicine, Kandahar University, Kandahar, Afghanistan; 2 Faculty of Medicine, Afghan International Islamic University, Kabul, Afghanistan; 3 Afghanistan National Public Health Institute, Ministry of Public Health, Kabul, Afghanistan; 4 Faculty of Education and Training, Kandahar University, Kandahar, Afghanistan; 5 Centre for Tropical Medicine and Global Health, Nuffield Department of Medicine, University of Oxford, Oxford, United Kingdom; 6 Mahidol Oxford Tropical Medicine Clinical Research Unit (MORU), Mahidol University, Bangkok, Thailand; PSMRI, INDIA

## Abstract

**Background:**

Cutaneous leishmaniasis (CL) is highly endemic in Kandahar province of Afghanistan, but data on the population’s knowledge of CL and measures they adopt to prevent it are unknown. The main objectives of the study were to study the knowledge, attitude, and prevention practices of CL, and their associated factors, in two highly-endemic rural districts, Daman and Arghandab, in Kandahar province.

**Methods:**

This community-based cross-sectional analytical study took place over seven months, from September 2024–March 2025, in adults. Data were analysed by descriptive statistics, the Chi-square test, and multivariate logistic regression.

**Results:**

A total of 2,118 adults were recruited with a mean age of 35.8 years; 60.3% were males, 56.1% farmers, 91.5% illiterate, and 91.4% from poor families. Among the study participants, 24.1%, 41.5%, and 17.9% had good CL knowledge, a positive attitude towards CL, and good preventive practices towards CL. Independent factors associated with: (i) poor CL knowledge were being not single (adjusted odds ratio [AOR] 1.2), being a farmer (AOR 1.1) and coming from a poor family (AOR 1.3), (ii) a negative attitude towards CL were being aged >40 years (AOR 1.3) female (AOR 1.5), a resident in Arghandab district (AOR 1.2), and literate (AOR 1.1), and (iii) poor preventive practices against CL were being resident of Daman district (AOR 1.6), single (AOR 1.5), and illiterate (AOR 2.5).

**Conclusions:**

The majority of Daman and Arghandab residents had poor CL knowledge, a negative attitude, and poor prevention practices. Our results underscore the need for the Afghan Ministry of Public Health and international donor agencies, such as WHO and UNICEF, to plan and implement strategies to create/increase awareness of CL and measures to prevent and control it in Kandahar Province and beyond.

## Author summary

Cutaneous leishmaniasis (CL) is a neglected tropical disease with an estimated 600,000–1,000,000 new cases reported annually. More than one billion people live in areas endemic for leishmaniasis, and in 2021, > 85% of new CL cases occurred in nine countries: Afghanistan, Algeria, Brazil, Colombia, Iran, Iraq, Pakistan, Peru, and Syria. In Afghanistan, CL is focally endemic within major cities such as Kabul, Herat, and Kandahar, and is associated strongly with war, poverty, international sanctions, population displacement, destruction of buildings, and health system deterioration. Several studies have shown that an effective method to control infectious diseases is to increase the knowledge and attitude of a community. To our knowledge, no knowledge, attitude, and practice (KAP) studies on CL have been conducted in Afghanistan. Therefore, we conducted a community-based KAP survey to ascertain factors associated with CL knowledge, attitudes towards it, and what measures communities take to prevent it in two highly-endemic, rural districts of Kandahar province, Afghanistan. We identified several factors associated with poor knowledge, attitude, and prevention practices, including coming from a poor small family, being a farmer, having low literacy, and a CL case in a family. These data reconfirm poverty and low education as associated factors for CL, as well as farmers from small families already affected by CL, emphasising the need for health education campaigns targeting key risk groups identified from this large study.

## Introduction

Cutaneous leishmaniasis (CL) is a neglected tropical disease (NTD) caused by a parasite of the genus Leishmania and transmitted by the bite of an infected female sandfly [1]. Globally, the WHO estimates that more than one billion people live in areas endemic for leishmaniasis and are at risk of infection [2]. Approximately 600,000–1,000,000 new cases of CL are reported every year [2], and 40 million people have inactive CL scars [3]. However, under-reporting grossly underestimates the true global incidence and burden [4]. CL is endemic in more than 90 countries, but some 80% of CL cases are reported from the Middle East and North Africa (MENA) region, especially Afghanistan, Algeria, Iran, Syria, Pakistan, Iraq, Yemen, and Saudi Arabia [4–8].

Clinically, CL starts with a skin lesion at the bite site of sandfly that usually increases in size to form a nodule that commonly ulcerates and may become secondarily infected by bacteria and/or fungi [9,10]. If left untreated, CL leaves life-long scars, resulting in disfigurement and social stigma [1,11]. The main factors associated with CL include poverty, young age, climate change, illiteracy, lack of preventive measures, migration, deforestation, malnutrition, as well as specific occupations and activities like farming, soldiering, mining, and hunting [1,12–15].

The successful control of CL rests on the adherence of both treatment and preventive measures; treatment adherence in the endemic areas is largely affected by the inhabitants’ knowledge about CL and the responsible vectors, as well as their attitudes towards CL [16]. Knowledge, attitude, and practices (KAP) surveys on CL have been conducted in several MENA countries, e.g., Pakistan [17], Iran [18], Saudi Arabia [19], Syria [20], Yemen [21], Algeria [22], and Morocco [23]. Moreover, several studies have shown that increasing the knowledge and attitudes of communities results in better control of infectious diseases [21].

CL is focally endemic in Afghanistan, especially in major cities such as Kabul, Herat, and Kandahar [24], where *L. tropica* is the predominant species and is characterised by human-to-human transmission [25,26]. To the best of our knowledge, no KAP studies on CL have been conducted in Afghanistan. Therefore, we set out to conduct a KAP survey and identify key factors to explain the current level of knowledge about CL in two highly-endemic rural districts of Kandahar province, Afghanistan.

## Methods

### Study design and study area

This was a community-based cross-sectional analytical study, conducted over seven months (September 2024–March 2025) in the rural districts of Daman and Arghandab in Kandahar province. Daman district is located 18 kilometres southeast of Kandahar city with a population of 54,688 inhabitants, and Arghandab district is located 9 kilometres to the north of Kandahar city and has a population of 71,514 people [27]. Both districts are entirely rural, and most residents are farmers. They are famous for their orchards of grapes, pomegranates, and plums, as well as the cultivation of wheat and corn.

### Study population and sample size calculation

Our source population was composed of only adults (>18 years old), both males and females, willing to participate in this study, and permanent residents of one of the two included districts of Kandahar province. Individuals were excluded from this study who did not consent to take part in this study, or were either returnees or internally displaced.

The sample size and power calculations were performed in Epi Info version 7.2 (CDC, Atlanta, Georgia, USA). The expected response frequency to a given question was chosen at 50%, with an acceptable margin of error of 3% and a confidence level of 99%. Adding a 15% non-response rate, the target sample size was 2088 individuals from the two districts combined.

### Ethical considerations

Prior to the study, written informed consent was obtained from all the study participants which included the voluntary nature of the study and measures taken to ensure confidentiality. Only participants’ initials were used on the case record form, and these were coded and de-identified before data entry. Identification information of the participants will not be disclosed. Ethical approval was taken from the Kandahar University Ethics Committee (code number KDRU-EC-2024.08). The study was conducted based on the Declaration of Helsinki, 2008.

### Sample selection & data collection

For data collection in each of the two selected districts, villages with ≥50 houses each were randomly selected using a lottery method. In each village, we selected only one adult per house using convenience sampling. The null hypothesis to be tested in this study was as follows: The knowledge, attitudes, and practices of the people regarding cutaneous leishmaniasis are adequate for effective prevention and control of CL in Daman and Arghandab districts of Kandahar province. The questionnaire was developed based on a literature review of similar studies in different parts of the world, as well as comments from the local Afghan experts of CL. It was first developed in English and translated into Pashto, the local language, and then pre-tested in non-selected individuals to assess content validity, appropriateness, and question comprehensibility. The questionnaire consisted of socio-demographic characteristics, knowledge about and attitude towards CL, including knowledge of the sandfly vector and measures taken to prevent CL (see below). In total, there were 43 questions in the questionnaire, i.e., 9 about sociodemographic information, 15 on knowledge about CL, 12 on attitude towards CL, and 8 questions on prevention practice towards CL.

### Definitions

#### Poverty.

Poverty was defined based on The World Bank definition, i.e., a family that earns <150 Afghanis (<2.15 USD) per person per day [28].

**Knowledge about CL:** This score was based on 15 questions: (a) identification of CL picture, (b) heard about CL, (c) ever had CL, (d) transmission of CL via mouse, (e) vector for transmission of CL, (f) sign(s) of CL, (g) location of CL lesions/scars, (h) habitat of the sandfly, (i) communicability of CL, (j) acquiring of CL in traveling to endemic areas, (k) biting time of the sandfly, (l) seriousness of CL, (m) preventability of CL, (n) prevention measures for CL, and (o) complete cure available. A score of 1 point was given for a correct response and 0 for an incorrect/don’t know response. Poor knowledge about CL was defined as a study participant who scored 0–7 points. Good knowledge about CL was defined as a study participant who scored 8–15 points.

**Attitude towards CL:** This was based on the 12-item questionnaire assessing overall attitude towards CL: (a) CL is a problem in the area, (b) CL is treatable, (c) untreated CL causes disability, (d) CL affects household economy, (e) high incidence season of CL, (f) CL transmission via direct contact, (g) the importance of environmental sanitation, (h) feeling well-informed about CL, (i) breeding places of the sandfly, (j) spirituality of CL, (k) a relation of CL with rodents, and (l) CL can cause anxiety. Answers for the attitude questions were designed with a five-point Likert scale, i.e., (a) strongly disagree, (b) disagree, (c) neutral, (d) agree, and (e) strongly agree. Later, a score of 1 point was given for a correct response and 0 for an incorrect/neutral response. Negative attitude about CL was defined as a study participant who scored 0–5 points. A positive attitude about CL was defined as a study participant who scored 6–12 points.

**Prevention practices towards CL:** There were 8 questions about prevention practices towards CL: (a) bed net use, (b) working time, (c) sleeping outdoors, (d) repellent utilization, (e) proper garbage disposal, (f) indoor residual spray in the last 12 months, (g) participation in CL control activities, and (h) preference of treatment method for CL. A score of 1 point was given for a correct response and 0 for an incorrect/don’t know response. Poor prevention practice towards CL was defined as a study participant who scored 0–3 points. Good prevention practice about CL was defined as a study participant who scored 4–8 points.

### Data analysis

Data were double-entered and cleaned by two independent data entry clerks in Microsoft Excel 2021 before analysis using Statistical Package for the Social Sciences (SPSS) version 22 (Chicago, IL, USA). Descriptive analysis, including frequency, percentage, mean, standard deviation (SD), and range, was used to summarise socio-demographic characteristics. The Chi-square test (using crude odds ratio [COR]) was performed to assess the association between categorical variables and CL knowledge, attitude towards CL, and prevention practices against CL. All variables that were statistically significant in univariate analyses were assessed for independence in a multivariable logistic regression model, using adjusted odds ratio (AOR). Potential explanatory factors were: age, sex, district of residence, marital status single vs. married/divorced/widow), occupation (farmer vs. non-farmer), literate vs. illiterate, poor vs. not poor, family size (<5 people vs. ≥ 5 people), and a confirmed CL case in the family. For all tests, a two-sided *p*-value of <0.05 was considered statistically significant.

## Results

A total of 2,183 individuals were approached, and 65 declined to participate, leaving 2,118 responders, 954 (45.0%) from Arghandab and 1,164 (55.0%) from Daman, for a response rate of 97.0%.

### Socio-demographic characteristics

The mean (SD) age of all participants was 35.8 (10.4) years, and 60.3% (1,277/2,118) were males. By occupation, 56.1% (1,189/2,118) were farmers, 91.5% (1,938/2,118) were illiterate, 91.4% (1,935/2,118) belonged to a poor family, while 31.6% (669/2,118) had a confirmed CL in a family member (Table 1).

**Table 1:** Socio-demographic and other characteristics of the study participants.

Variable	Total, n (%) (n = 2118)	Study location (district)	
		Daman, n (%) (n = 1164)	Arghandab, n (%) (n = 954)
Age			
18–40 years	1603 (75.7)	875 (75.2)	728 (76.3)
> 40 years	515 (24.3)	289 (24.8)	226 (23.7)
Gender			
Male	1277 (60.3)	756 (64.9)	521 (54.6)
Female	841 (39.7)	408 (35.1)	433 (45.4)
Marital status			
Single	372 (17.6)	181 (15.5)	191 (20.0)
Married/Divorced/Widow	1746 (82.4)	983 (84.5)	763 (80.0)
Occupation			
Farmer	1189 (56.1)	502 (43.1)	687 (72.0)
Non-farmer*	929 (43.9)	662 (56.9)	267 (28.0)
Literacy level			
Literate	180 (8.5)	104 (8.9)	76 (8.0)
Illiterate	1938 (91.5)	1060 (91.1)	878 (92.0)
Family economic status			
Poor	1935 (91.4)	1060 (91.1)	875 (91.7)
Not poor	183 (8.6)	104 (8.9)	79 (8.3)
Family size			
< 5 people	486 (22.9)	271 (23.3)	215 (22.5)
≥ 5 people	1632 (77.1)	893 (76.7)	739 (77.5)
Presence of confirmed CL case among family members			
Yes	669 (31.6)	369 (31.7)	300 (31.4)
No	1449 (68.4)	795 (68.3)	654 (68.6)

CL, Cutaneous Leishmaniasis; n, Number.

*Non-farmers included housewives (754), shopkeepers (46), employees (21), and jobless (108).

### Knowledge about CL

Although a majority of the participants (86.8% or 1,839/2,118) had heard about CL, 75.9% (1,608/2118) had poor CL knowledge. Among the study participants, 0%, 0.4%, and 7.2% knew that CL is transmitted by sandfly, or rodents, and that CL is a preventable disease, respectively (Table 2).

**Table 2:** Knowledge of the study participants about cutaneous leishmaniasis in Daman and Arghandab districts of Kandahar Province (n = 2118).

Variable	Frequency (n)	Percentage (%)
Able to identify as CL	847	40.0
Unable to identify as CL	1271	60.0
Have you heard about CL?		
Yes	1839	86.8
No	279	13.2
Have you ever got CL?		
Yes	460	21.7
No	1658	78.3
Is CL transmitted by rodents?		
Yes	9	0.4
No	340	16.1
I don’t know	1769	83.5
Vector for transmission of CL		
Sandfly	0	0.0
Mosquito	184	8.7
I don’t know	1934	91.3
Is skin lesion the sign of CL?		
Yes	316	14.9
No	264	12.5
I don’t know	1538	72.6
Are face, arm, leg, and ear the body parts for the location of CL lesion/scar?		
Yes	237	11.2
No	173	8.2
I don’t know	1708	80.6
Are rock crevices, caves, rodent burrows, and vegetation the habitats of sandfly?		
Yes	8	0.4
No	489	23.1
I don’t know	1621	76.5
Is CL transmitted from an infected to a healthy person?		
Yes	282	13.3
No	521	24.6
I don’t know	1315	62.1
Can CL be acquired by travelling to endemic areas?		
Yes	119	5.6
No	386	18.2
I don’t know	1613	76.2
Are dawn and dusk the preferred biting times of the vector?		
Yes	123	5.8
No	1479	69.8
I don’t know	516	24.4
Is CL a serious disease?		
Yes	80	3.8
No	843	39.8
I don’t know	1195	56.4
Is CL a preventable disease?		
Yes	152	7.2
No	594	28.0
I don’t know	1372	64.8
Are health education, hygiene, and sanitation the preventive measures of CL?		
Yes	214	10.1
No	385	18.2
I don’t know	1519	71.7
Is there complete cure from CL?		
Yes	159	7.5
No	1853	87.5
I don’t know	106	5.0
**Overall knowledge about CL**		
Poor knowledge (score 0–7)	1608 (75.9%)	
Good knowledge (score 8–15)	510 (24.1%)	

CL, Cutaneous Leishmaniasis; n, Number.

### Attitude towards CL

More than half, 58.5% (n = 1,239), had a negative attitude towards the CL. Although a sizable minority, 43.9% (n = 930), agreed/strongly agreed that CL is a health problem in their area, only 27.3% (n = 578) and 22.6% (n = 478) reported that CL is transmitted by direct contact from person to person and is a spiritual disease, respectively. Moreover, 1,823 (86.1%) disagreed/strongly disagreed that they thought they were well informed about CL, and 854 (40.3%) disagreed/strongly disagreed that environmental sanitation is important for the prevention of CL transmission (Table 3).

**Table 3:** The attitude of the study participants towards CL in Daman and Arghandab districts of Kandahar Province (n = 2118).

Variable	Measurements for attitude towards CL				
	Strongly agree, n (%)	Agree, n (%)	Neutral, n (%)	Disagree, n (%)	Strongly disagree, n (%)
CL is a health problem in the area	663 (31.3)	267 (12.6)	582 (27.5)	477 (22.5)	129 (6.1)
CL can be treated	180 (8.5)	159 (7.5)	926 (43.7)	773 (36.5)	80 (3.8)
If not treated earlier, disability is the outcome of CL	146 (6.9)	100 (4.7)	1006 (47.5)	779 (36.8)	87 (4.1)
CL in one family member affects the economy of the whole family	9 (0.4)	5 (0.2)	1455 (68.7)	521 (24.6)	128 (6.0)
Highest CL incidence is in autumn season	121 (5.7)	155 (7.3)	1563 (73.8)	146 (6.9)	133 (6.3)
CL is transmitted by direct contact from person to person	321 (15.2)	257 (12.1)	1437 (67.8)	86 (4.1)	17 (0.8)
Environmental sanitation is important for the prevention of CL transmission	152 (7.2)	114 (5.4)	998 (47.1)	436 (20.6)	418 (19.7)
You think you are well informed about CL	8 (0.4)	43 (2.0)	244 (11.5)	931 (44.0)	892 (42.1)
Vegetation area, rock cracks, and animal manures are the major breeding places of sandfly	121 (5.7)	210 (9.9)	1008 (47.6)	357 (16.9)	422 (19.9)
CL is a spiritual disease	203 (9.6)	275 (13.0)	974 (46.0)	621 (29.3)	45 (2.1)
CL has a relationship with rodents	0 (0.0)	0 (0.0)	1745 (82.4)	316 (14.9)	57 (2.7)
CL can cause anxiety	74 (3.5)	64 (3.0)	1924 (90.8)	35 (1.7)	21 (1.0)
**Overall attitude status**					
Negative attitude (score 0–5)	1239 (58.5%)				
Positive attitude (score 6–12)	879 (41.5%)				

CL, Cutaneous Leishmaniasis; n, Number.

### Prevention practices towards CL

Poor prevention practice towards CL was reported by 82.1% (n = 1,739) of the study participants. Only 4.8% (n = 102) properly disposed of garbage, 10.8% (n = 229) used repellents, but 45.3% (n = 959) used bed nets (Table 4).

**Table 4:** The prevention practices of the study participants towards the CL in Daman and Arghandab districts of Kandahar Province (n = 2118).

Variable	Frequency (n)	Percentage (%)
Yes	959	45.3
No	1159	54.7
Working time		
Day	1923	90.8
Night	49	2.3
Both day and night	146	6.9
Sleeping outdoors		
No	57	2.7
Yes	2061	97.3
Use of repellents for CL prevention		
Yes	229	10.8
No	1889	89.2
Properly performing garbage disposal		
Yes	102	4.8
No	2016	95.2
Indoor residual spray in the last 12 months		
Yes	182	8.6
No	1936	91.4
Ever participated in CL control activities		
Yes	4	0.2
No	2114	99.8
CL treatment method preference		
Modern treatment	222	10.5
Traditional treatment	707	33.4
Both modern and traditional treatments	1189	56.1
**Overall prevention practices towards CL**		
Poor prevention practices (score 0–3)	1739 (82.1%)	
Good prevention practices (score 4–8)	379 (17.9%)	

CL, Cutaneous Leishmaniasis; n, Number.

### Factors associated with poor knowledge about CL

Of the nine assessed variables, three were found to be statistically significant in the univariate and multivariable analyses (Table 5): being married/divorced/widow (COR 1.1, AOR 1.2), being a farmer (COR 1.3, AOR 1.1), and belonging to a poor family (COR 1.1, AOR 1.3).

**Table 5:** Univariate analyses of the factors associated with knowledge about CL of the study participants.

Variable	Knowledge about CL					
	Poor n (%), n=1608	Good n (%), n=510	COR (95% CI)	*p*-value	AOR (95% CI)**	*p*-value
Age				0.058	–	–
18–40 years	1201 (74.9)	402 (25.1)	1			
> 40 years	407 (79.0)	108 (21.0)	1.2 (1.0–1.4)			
Gender				0.132	–	–
Male	955 (74.8)	322 (25.2)	1			
Female	653 (77.6)	188 (22.4)	1.1 (1.0–1.3)			
Residence				0.373	–	–
Daman	875 (75.2)	289 (24.8)	1			
Arghandab	733 (76.8)	221 (23.2)	1.1 (0.9–1.2)			
Marital status				0.002		0.002
Single	259 (69.6)	113 (30.4)	1		1	
Married/Divorced/ Widow	1349 (77.3)	397 (22.7)	1.1 (1.0–1.1)		1.2 (1.1–1.3)	
Occupation				<0.001		<0.001
Farmer	957 (80.5)	232 (19.5)	1		1	
Non-farmer*	651 (70.1)	278 (29.9)	1.3 (1.2–1.5)		1.1 (1.0–1.2)	
Literacy level				0.181	–	–
Literate	144 (80.0)	36 (20.0)	1.3 (0.9–1.8)			
Illiterate	1464 (75.5)	474 (24.5)	1			
Family economic status				<0.001		0.001
Poor	1489 (77.0)	446 (23.0)	1		1	
Not poor	119 (65.0)	64 (35.0)	1.1 (1.0–1.2)		1.3 (1.2–1.5)	
Family size				0.335	–	–
< 5 people	361 (74.3)	125 (25.7)	1.0 (1.0–1.1)			
≥ 5 people	1247 (76.4)	385 (23.6)	1			
Presence of confirmed CL case among family members				0.387	–	–
Yes	500 (74.7)	169 (25.3)	1			
No	1108 (76.5)	341 (23.5)	1.0 (1.0–1.1)			

AOR, Adjusted Odds Ratio; CL, Cutaneous Leishmaniasis; CI, Confidence Interval; COR, Crude Odds Ratio; n, number.

*Non-farmers included housewives (754), shopkeepers (46), employees (21), and jobless (108).

**Variables with non-significance in Chi-square analysis were not included in the logistic regression model.

### Factors associated with a negative attitude towards CL

Similarly, five and four variables were associated with a negative CL attitude in the univariate and multivariable analyses, respectively (Table 6): aged >40 years (COR 2.0, AOR 1.3), being female (COR 1.4, AOR 1.5), a resident of Arghandab district (COR 1.5, AOR 1.2), and being literate (COR 2.0, AOR 1.1).

**Table 6:** Univariate analyses of the factors associated with the attitude towards CL of the study participants.

Variable	Attitude towards CL					
	Negative n (%), n=1239	Positive n (%), n=879	COR (95% CI)	*p*-value	AOR (95% CI)**	*p*-value
Age				<0.001		<0.001
18–40 years	859 (53.6)	744 (46.4)	1		1	
> 40 years	380 (73.8)	135 (26.2)	2.0 (1.7–2.4)		1.3 (1.2–1.4)	
Gender				<0.001		<0.001
Male	687 (53.8)	590 (46.2)	1		1	
Female	552 (65.6)	289 (34.4)	1.4 (1.2–1.5)		1.5 (1.2–1.8)	
Residence				<0.001		<0.001
Daman	598 (51.4)	566 (48.6)	1		1	
Arghandab	641 (67.2)	313 (32.8)	1.5 (1.3–1.6)		1.2 (1.1–1.2)	
Marital status				0.219	–	–
Single	207 (55.6)	165 (44.4)	1			
Married/Divorced/ Widow	1032 (59.1)	714 (40.9)	1.0 (1.0–1.1)			
Occupation				<0.001		0.091
Farmer	776 (65.3)	413 (34.7)	1		1	
Non-farmer*	463 (49.8)	466 (50.2)	1.3 (1.2–1.4)		0.6 (0.5–0.7)	
Literacy level				<0.001		<0.001
Literate	133 (73.9)	47 (26.1)	2.0 (1.5–2.8)		1.1 (1.0–1.2)	
Illiterate	1106 (57.1)	832 (42.9)	1		1	
Family economic status				0.115	–	–
Poor	1142 (59.0)	793 (41.0)	1			
Not poor	97 (53.0)	86 (47.0)	1.0 (1.0–1.0)			
Family size				0.578	–	–
< 5 people	279 (57.4)	207 (42.6)	1.0 (1.0–1.1)			
≥ 5 people	960 (58.8)	672 (41.2)	1			
Presence of confirmed CL case among family members				0.659	–	–
Yes	396 (59.2)	273 (40.8)	1			
No	843 (58.2)	606 (41.8)	1.0 (0.9–1.2)			

AOR, Adjusted Odds Ratio; CL, Cutaneous Leishmaniasis; CI, Confidence Interval; COR, Crude Odds Ratio; n, number.

*Non-farmers included housewives (754), shopkeepers (46), employees (21), and jobless (108).

**Variables with non-significance in Chi-square analysis were not included in the logistic regression model.

### Factors associated with poor preventive practices

Poor preventive CL practices were independently associated (Table 7) with being resident of Daman (COR 1.5, AOR 1.6), single (COR 1.5, AOR 1.5), and illiterate (COR 2.5, AOR 2.5).

**Table 7:** Univariate analyses of the factors associated with prevention practices of the study participants.

Variable	Prevention practices against CL					
	Poor n (%), n=1739	Good n (%), n=379	COR (95% CI)	*p*-value	AOR (95% CI)**	*p*-value
Age				0.807	–	–
18–40 years	1318 (82.2)	285 (17.8)	1			
> 40 years	421 (81.7)	94 (18.3)	1.0 (0.9–1.1)			
Gender				0.384	–	–
Male	1056 (82.7)	221 (17.3)	1			
Female	683 (81.2)	158 (18.8)	1.0 (0.9–1.1)			
Residence				<0.001		<0.001
Daman	989 (85.0)	175 (15.0)	1		1	
Arghandab	750 (78.6)	204 (21.4)	1.5 (1.2–1.9)		1.6 (1.2–1.9)	
Marital status				0.014		0.013
Single	322 (86.6)	50 (13.4)	1		1	
Married/Divorced/ Widow	1417 (81.2)	329 (18.8)	1.5 (1.1–2.1)		1.5 (1.1–2.1)	
Occupation				0.347	–	–
Farmer	968 (81.4)	221 (18.6)	1			
Non-farmer*	771 (83.0)	158 (17.0)	1.1 (0.9–1.4)			
Literacy level				<0.001		<0.001
Literate	121 (67.2)	59 (32.8)	2.5 (1.8–3.4)		2.5 (1.8–3.5)	
Illiterate	1618 (83.5)	320 (16.5)	1		1	
Family economic status				0.800	–	–
Poor	1590 (82.2)	345 (17.8)	1			
Not poor	149 (81.4)	34 (18.6)	1.0 (1.0–1.0)			
Family size				0.279	–	–
< 5 people	391 (80.5)	95 (19.5)	1.0 (1.0–1.1)			
≥ 5 people	1348 (82.6)	284 (17.4)	1			
Presence of confirmed CL case among family members				0.688	–	–
Yes	546 (81.6)	123 (18.4)	1			
No	1193 (82.3)	256 (17.7)	1.0 (0.9–1.1)			

AOR, Adjusted Odds Ratio; CL, Cutaneous Leishmaniasis; CI, Confidence Interval; COR, Crude Odds Ratio; n, number.

*Non-farmers included housewives (754), shopkeepers (46), employees (21), and jobless (108).

**Variables with non-significance in Chi-square analysis were not included in the logistic regression model.

## Discussion

In this large cross-sectional analytical community-based study of adult rural dwellers, less than a quarter of the study participants had a good knowledge of CL, less than half had a positive attitude towards CL, and only a small minority were taking preventative measures towards CL.

To our knowledge, this is the first KAP survey to have been conducted in rural areas of Afghanistan, a country with one of the world’s largest CL burden. The large sample size gave us statistical power to assess nine potential explanatory factors. Poor knowledge was associated with poverty and being a farmer who also did not use prevention methods. Being illiterate and coming from a small family were associated with a poor attitude. Men were found to have a better attitude than women, but were worse than women at trying to prevent CL. Our findings compare and contrast with other work.

A recent study conducted in Kandahar city among urban adults revealed that 23.6%, 40.6%, and 33.3% of the study participants had good knowledge of CL, a positive attitude towards CL, and practiced preventive measures against CL, respectively [29].

In Ethiopia, almost half of the 612 people had good knowledge about CL, two-fold higher than in our study; poor knowledge was associated with illiteracy, being poor (consistent with our findings), not using media, and not knowing someone with CL [30]. Consistent findings were reported from Yemen, where just over half of 289 households had good CL knowledge. Similar to our findings, all study participants had heard of CL, but just under 10% (27/289) could say that it is caused by sandflies, compared to our 7% [9]. Yemeni farmers were also identified as a key group with poor knowledge, and in contrast to us, younger females from families without a CL case were a factor for poor knowledge. Studies from Khyber Pakhtunkhwa province, which borders Afghanistan, and Quetta (230 km from our study areas in Afghanistan) shed light on regions where *L. tropica* is common [17,31].

Consistent with Kandahar, overall CL knowledge was low in both places: ~ 27% in Khyber Pakhtunkhwa and ~37% in Quetta [31], but there was contrasting knowledge regarding sand flies.

About 59% of Quetta [31] residents knew sand flies were the vectors compared to ~16% in Khyber Pakhtunkhwa and 0% in Kandahar. Almost no one in Kandahar thought rodents were an important reservoir, whilst 13% identified humans as a source of infection, some four-fold lower than in Quetta (47%) [31]. Some 40% of our residents thought CL was not a serious disease (with 55% not knowing), similar to the 37% from Quetta [31], and only 7% thought CL was preventable compared to ~27% in Yemen [9].

A little more than one-third of Khyber Pakhtunkhwa residents had seen a case of CL. Although not asked in our survey, ~ 20% of our population had had CL. In Quetta, 42% reported that CL rates were higher in winter [31]; the latter is typical of *L. tropica* [32]. Inter-study differences could be attributed to the sociocultural differences, levels of education, access to media, and provision of health education.

In our study, less than half had a positive attitude towards the CL, less than the 55% reported from Ethiopia [30], but more than the 38% of Yemeni household heads [9]. Associated factors in Yemen were resident in the Razeh area of Yemen and the presence of confirmed CL cases in the family members [9], whilst a higher age (>54.5 years) and visiting traditional healers were important in Ethiopia [30]. However, only age > 40 years was identified in our study, again reflecting different CL settings. Most Quetta residents (80%) thought CL was a problem in their city [31] compared to 44% of our rural residents, reflecting the urban predominance of *L. tropica*.

A study conducted in Quetta, Pakistan, among suspected or confirmed cases of CL revealed that 80% of the study participants thought that CL is a major public health issue in Quetta, 37% did not think that CL is dangerous, 88% agreed that CL causes social discomfort due to its disfigurement, while 47% believed that living with CL infected person increases the risk of getting CL [31].

A cross-sectional study conducted among 844 rural people in Khyber Pakhtunkhwa province of Pakistan revealed that 99.2% of the study participants had wild animal reservoirs in the close vicinity and 97.0% people had domestic animals in the household [17].

In our study, only 17.9% of the study participants had good prevention practices. The statistically significant factors associated with poor preventive practices towards CL were being a resident of Daman district, single, and illiterate. CL control activities are rarely implemented in these areas; only four of our study participants reported participating in them. However, some residents apply the IRS without any technical support from experts. Residents in these study areas typically purchase insecticides over the counter and apply them independently, largely for controlling other dangerous insects such as scorpions and poisonous spiders. An Ethiopian study reported that 35.3% of the study population had good prevention practices towards CL. The statistically significant factors associated with poor prevention practices towards CL were being male, age < 44.5 years, and not knowing someone with CL [30].

A cross-sectional study conducted among 844 rural people in Khyber Pakhtunkhwa province of Pakistan revealed that 82.8% of the study participants were using mosquito nets while sleeping, 85.3% were using insecticide spray in the household, and 80.0% were using mosquito repellents [17]. A community-based cross-sectional study conducted among 289 household heads in rural Yemen reported that 16.3% of them had good prevention practices towards CL, with 9.0% and 9.3% of the study participants using bed nets and using insecticide spray in the household, respectively. The statistically significant factor associated with poor prevention practices was having a low income [9]. A study conducted in Quetta, Pakistan, among suspected or confirmed cases of CL reported that 94% of the study participants sought modern medical care for the treatment of CL, 89% were using bed nets that were not insecticide-treated, 61% were not using any insect repellents, while 24% of them were not aware of CL preventive methods [31].

The percentage of good prevention practices observed in our study was less than that of the studies mentioned above. The variation in the prevention of good prevention practices and its associated factors in different countries could be due to the variations in health education, literacy rate, weather, economic status, and culture of the people.

### Limitations

There were several limitations in our study. As a cross-sectional survey, we were only able to capture a snapshot of KAPs at one point in time. A longitudinal study design would have been better to observe trends over time. Second, we relied on self-reported data that could introduce recall and social desirability biases, especially regarding sensitive topics like stigma and mental health. Data were collected from two rural districts where the leishmania species is unknown. Such settings tend to harbour *L. major*. However, given the agricultural richness, *L. tropica* is likely to be a significant contributor to CL cases [25,33]. Nevertheless, we cannot generalise these data to the whole population of Afghanistan. Third, the definition of poverty was based on a single question: family income per person per day.

## Conclusion

We have identified significant gaps in CL knowledge, which went hand in hand with a negative attitude towards CL and poor prevention practices. Independent factors associated with: (i) poor CL knowledge were being not single, being a farmer and coming from a poor family, (ii) a negative attitude towards CL were being aged >40 years, female, a resident in Arghandab district, and literate, and (iii) poor preventive practices against CL were being resident of Daman district, single, and illiterate. These findings can serve as the basis for health education campaigns on CL, with special attention given to poor illiterate families and farmers. Such a programme could be part of a comprehensive CL control strategy formulated by policy-makers, healthcare planners, working with, e.g., the WHO and UNICEF. More research is needed in other CL settings in Afghanistan, like the densely populated cities and arid rural regions.

## Supporting information

S1 File SPSS file of CL KAP study. (SAV)
