# Supplementary material for: Knowledge, attitude, and prevention practices of cutaneous leishmaniasis in highly-endemic rural areas of Kandahar province, Afghanistan: A large cross-sectional community-based study
Source: PLoS Negl Trop Dis. 2026 Jun 3;20(6):e0013575. doi: 10.1371/journal.pntd.0013575 (PMC13245870; doi:10.1371/journal.pntd.0013575)
Supplement: Attachment — Submitted filename: Response to reviewers.docx [file pntd.0013575.s002.docx]

**Journal Requirements:**

If the reviewer comments include a recommendation to cite specific previously published works, please review and evaluate these publications to determine whether they are relevant and should be cited. There is no requirement to cite these works unless the editor has indicated otherwise.

1) Please upload all main figures as separate Figure files in .tif or .eps format. For more information about how to convert and format your figure files please see our guidelines: 
<https://journals.plos.org/plosntds/s/figures>

**Answer:** Many thanks. The figure (map) we used was not crucial. So, we removed it.

2) Some material included in your submission may be copyrighted. According to PLOSu2019s copyright policy, authors who use figures or other material (e.g., graphics, clipart, maps) from another author or copyright holder must demonstrate or obtain permission to publish this material under the Creative Commons Attribution 4.0 International (CC BY 4.0) License used by PLOS journals. Please closely review the details of PLOSu2019s copyright requirements here: PLOS Licenses and Copyright. If you need to request permissions from a copyright holder, you may use PLOS's Copyright Content Permission form.
Please respond directly to this email and provide any known details concerning your material's license terms and permissions required for reuse, even if you have not yet obtained copyright permissions or are unsure of your material's copyright compatibility. Once you have responded and addressed all other outstanding technical requirements, you may resubmit your manuscript within Editorial Manager.

**Answer:** Thanks. The figure (map) we used was not crucial. So, we removed it.

Potential Copyright Issues:
i) Figure 1. Please (a) provide a direct link to the base layer of the map (i.e., the country or region border shape) and ensure this is also included in the figure legend; and (b) provide a link to the terms of use / license information for the base layer image or shapefile. We cannot publish proprietary or copyrighted maps (e.g. Google Maps, Mapquest) and the terms of use for your map base layer must be compatible with our CC BY 4.0 license.
Note: if you created the map in a software program like R or ArcGIS, please locate and indicate the source of the basemap shapefile onto which data has been plotted.
If your map was obtained from a copyrighted source please amend the figure so that the base map used is from an openly available source. Alternatively, please provide explicit written permission from the copyright holder granting you the right to publish the material under our CC BY 4.0 license.
If you are unsure whether you can use a map or not, please do reach out and we will be able to help you. The following websites are good examples of where you can source open access or public domain maps:
* U.S. Geological Survey (USGS) - All maps are in the public domain. (<http://www.usgs.gov>)
* PlaniGlobe - All maps are published under a Creative Commons license so please cite u201cPlaniGlobe, <http://www.planiglobe.com>, CC BY 2.0u201d in the image credit after the caption. (<http://www.planiglobe.com/?lang=enl>)
* Natural Earth - All maps are public domain. (<http://www.naturalearthdata.com/about/terms-of-use/>).

**Answer:** Many thanks. The figure (map) we used was not crucial. So, we removed it.

**Reviewers' comments:**

Reviewer's Responses to Questions

**Key Review Criteria Required for Acceptance?**
As you describe the new analyses required for acceptance, please consider the following:

**Methods**
-Are the objectives of the study clearly articulated with a clear testable hypothesis stated?
-Is the study design appropriate to address the stated objectives?
-Is the population clearly described and appropriate for the hypothesis being tested?
-Is the sample size sufficient to ensure adequate power to address the hypothesis being tested?
-Were correct statistical analysis used to support conclusions?
-Are there concerns about ethical or regulatory requirements being met?

Reviewer #1: The methods are generally clear. There is no testable hypothesis, though as a descriptive work, the design is appropriate.
I had a couple of questions about findings that might be related to methods, or results. I will mention them here.
1) Line 279- The overall rate of 14.8% of study participants had a score classified as "good knowledge" of CL. But in the methods section (line 184) the definition of "good knowledge" is defined as the mean value on the survey instrument. This would seem to imply that necessarily 50% should have had good knowledge. If the method for this determination, for example taking the mean from some other validation or population, was used, please clarify.

**Answer:** Many thanks for the comment. Knowledge about CL was based on the mean score of the 14 knowledge measurement questions. Good and poor knowledge about CL were defined as scores of ≥8.4 and <8.4, respectively. These cut-off points were also used in study conducted by Geto et al in Ethiopia (doi:10.1371/journal.pntd.0012427).

2) Statistical analysis- It is striking that so many confidence intervals include or barely exclude 1, but the corresponding p values are highly significant. It is possible that this is from rounding of log ORs. It would be helpful the authors could comment.

**Answer:** Many thanks for the useful comment. We did the data analysis again. In the three variables where the p-values were highly significant, the CI values included 1. It has now been updated and revised.

Reviewer #2: The objectives of the study are clearly outlined. However, for improved clarity, it would be helpful to include the specific hypothesis that was tested. The study design appears appropriate.

**Answer:** Many thanks. The null hypothesis to be tested in this study was as follows: The knowledge, attitudes, and practices of the people regarding cutaneous leishmaniasis are adequate for effective prevention and control of CL in Daman and Arghandab districts of Kandahar province.

The section under "Study Population and Sample Size Calculation" should be reworded for enhanced clarity. Specifically, the inclusion and exclusion criteria need to be clearly stated. For instance, did the study exclude individuals aged '18 years or younger'? Was the study population drawn from all residents of the two districts, or only those living in the rural areas, as mentioned in line 152? If the latter, how were these ‘rural areas’ defined or selected?

**Answer:** Many thanks for the important comment. Now we have made things clear. Yes, only those >18 years old were included; ≤18 years were excluded. The two districts, where the study was conducted, were 100% rural areas. There were no urban areas. So, the study population was drawn from all the residents of the two districts.

Additionally, please specify the variables used in your sample size calculation. Was the final calculated sample size 2183?

**Answer:** Many thanks for the comment. The sample size and power calculations were performed in Epi Info version 7.2 (CDC, Atlanta, Georgia, USA). Expected frequency was chosen as 50%, an acceptable margin of error of 3%, and a confidence level of 99%. A 15% non-response rate was added. Our sample size was 2088 people living in the rural areas of the two districts. During the study period, 65 refused to participate in the study. Finally, we collected data from 2118 people (1008 from Arghandab and 1110 from Daman districts), with a response rate of 96.9%.

Lastly, in line 158, if the authors intend to refer to ‘identification information,’ it would be clearer to explicitly use the term “identification information” rather than just “information.”

**Answer:** Many thanks. Now just “information” is changed to “identification information”.

**Results**


-Does the analysis presented match the analysis plan?

-Are the results clearly and completely presented?

-Are the figures (Tables, Images) of sufficient quality for clarity?

Reviewer #1: The results are clearly presented.
The discussion would benefit from restricting somewhat. Currently there are long stretches of reciting extensive results from other studies that may or may not have relevance here. I would suggest selecting which results the authors wish to present and framing them as comparisons or contrasts to the results found in this survey.

**Answer:** Many thanks. Now the discussion section is revised as per the comment.

Reviewer #2: The results are presented clearly and concisely

**Conclusions**


-Are the conclusions supported by the data presented?

-Are the limitations of analysis clearly described?

-Do the authors discuss how these data can be helpful to advance our understanding of the topic under study?

-Is public health relevance addressed?

Reviewer #1: (No Response)

Reviewer #2: In lines 357-359, the statement, “Based on the results of our study, the majority of the rural population of Daman and Arghandab districts of Kandahar province in Afghanistan had poor knowledge about CL, a negative attitude towards CL, and poor prevention practices towards CL,” should more clearly specify the study population. As noted in a previous comment, it is important to clarify whether this refers to the entire rural population of Daman and Arghandab districts or to the broader population of both districts.

**Answer:** Many thanks for the comment. The Daman and Arghandab districts of Kandahar province have only rural areas. No urban population lives in these two districts.

Now the conclusion section is revised and made clear.

Additionally, it would enhance the clarity of the conclusion if you briefly highlight, at the beginning of the section, the statistically significant factors associated with your findings. This would provide readers with a clearer summary of the key takeaways from your study.

**Answer:** Many thanks. Now we have added the statistically significant factors associated with our findings at the beginning of the conclusion section.

**Editorial and Data Presentation Modifications?**


Use this section for editorial suggestions as well as relatively minor modifications of existing data that would enhance clarity. If the only modifications needed are minor and/or editorial, you may wish to recommend “Minor Revision” or “Accept”.

Reviewer #1: (No Response)

Reviewer #2: The manuscript would benefit from language editing to improve clarity and readability in several sections.

**Summary and General Comments**


Use this section to provide overall comments, discuss strengths/weaknesses of the study, novelty, significance, general execution and scholarship. You may also include additional comments for the author, including concerns about dual publication, research ethics, or publication ethics. If requesting major revision, please articulate the new experiments that are needed.

Reviewer #1: (No Response)

Reviewer #2: The authors have made a good effort in bridging a gap in available data related to leishmaniasis in a selected region in Afghanistan. Attending to the minor revisions mentioned will improve the manuscript.

**Answer:**  Many thanks
