# Supplementary material for: Knowledge, attitude, and prevention practices of cutaneous leishmaniasis in highly-endemic rural areas of Kandahar province, Afghanistan: A large cross-sectional community-based study
Source: PLoS Negl Trop Dis. 2026 Jun 3;20(6):e0013575. doi: 10.1371/journal.pntd.0013575 (PMC13245870; doi:10.1371/journal.pntd.0013575)
Supplement: Attachment — Submitted filename: Response_to_reviewers_auresp_2.docx [file pntd.0013575.s003.docx]

**Journal Requirements:**

Dear Dr. Rahimi,

**Additional Editor Comments:**

We thank you for your efforts to respond to the reviewers' comments with reference to your submitted manuscript.

We request your further attention to the following:

**a.** Selection of cut-offs for KAP scores: It appears that you have probably used a questionnaire in your study which is identical to that used in an Ethiopian study published in 2024 (Geto et al). You also state in your response to one of the reviewers that you used the same cut-offs for separating good and poor KAP scores as were used in the Geto et al paper. At the same time, in the Definitions section, you state that the cut-offs (8.4, 30.86, and 5.0) were the mean values of the respective K, A, P scores. By default it will be interpreted that these are mean scores from your study. It is virtually impossible for mean values from two different studies to be the same, even if they used identical tools.

**Answer**: Many thanks for the important comment. Based on your comment, now we have completely revised the KAP cut-off values for the manuscript. We used the cut-off values for this study based on the number of questions asked and their answers in our research. We have now defined these cut-off values in the Materials and Methods sections of our manuscript as follows:

- For knowledge, there were 15 questions. Scores were 0–15. So, the maximum score was 15. Poor knowledge about CL was defined as a study participant who scored 0–7 points. Good knowledge about CL was defined as a study participant who scored 8–15 points.
- For attitude, there were 12 questions. Scores were 0–12. So, the maximum score was 12. Negative attitude about CL was defined as a study participant who scored 0–5 points. A positive attitude about CL was defined as a study participant who scored 6–12 points.
- For practice, there were 8 questions. Scores were 0–8. So, the maximum score was 8. Poor prevention practice towards CL was defined as a study participant who scored 0–3 points. Good prevention practice about CL was defined as a study participant who scored 4–8 points.

**a1.** Could you confirm that you have used the cut-offs from the Geto et al paper, and that the cut-offs are not the mean scores from your study? If this is true, please state clearly in the Definitions sections that the cut-offs used are the same as in the in the Geto et al study, and provide the reason/justification for using the same cut-offs. For instance, you can say that you used the same cut-offs to make comparisons easier.

**Answer**: Many thanks. the cut-offs for KAP in this manuscript are now revised based on the context of Afghanistan. We defined these cut-off values in the Materials and methods sections of our manuscript.

**a2.** If the cut-offs are not the means of the scores from your study data, and since the main purpose of the paper is to provide descriptive analysis, it would be appropriate to provide the basic descriptive figures for at least the summary K, A, P scores - mean, median, mode, in the respective tables or in the Results section. This will help the reader understand the distribution of response scores more clearly.

**Answer**: Many thanks. Now we revised and defined the KAP cut-off values in the Materials and methods sections of our manuscript. Now the values are clear and easy to understand for the readers.

**b.** The Attitude questions are based on Likert scales. It has not been explained how the responses were scored using the Likert scales:
**b1.** Which questions scored high for 'strongly agree' or for 'strongly disagree', for instance?

**Answer**: Many thanks for the important point. Now the information has been made clear as follows:

Answers for the attitude questions were designed with a five-point Likert scale, i.e., (a) strongly disagree, (b) disagree, (c) neutral, (d) agree, and (e) strongly agree. Later, a score of 1 point was given for a correct response and 0 for an incorrect/neutral response. Negative attitude about CL was defined as a study participant who scored 0–5 points. A positive attitude about CL was defined as a study participant who scored 6–12 points.

Also, the questions that scored high for strongly agree/agree or strongly disagree/disagree has been added in the “Results” section.

**b2.** What was the maximum possible score?
**Answer**: Many thanks. All the cut-off values and analyses have been thoroughly revised in the entire manuscript. Now the cut-offs have been revised and made easier as follows:

For knowledge, there were 15 questions. Scores were 0–15. So, the maximum score was 15.

For attitude, there were 12 questions. Scores were 0–12. So, the maximum score was 12.

For practice, there were 8 questions. Scores were 0–8. So, the maximum score was 8.

**c. Table 1:** percentages are either wrongly calculated or labelled: The percentages are correctly calculated for the column 'Total', but are differently calculated for the columns 'Daman' and 'Arghandab': here, each cell carries row percentages, but the column label indicates that they are column percentages. In this situation, column percentages will make more useful sense than row percentages. The column labels are fine, just change the cell percentages to row percentages for the two district specific columns. This will make it easier for the reader to judge the differences between the two districts.

**Answer**: OK, many thanks. Now the percentages have been changed from rows to column.


**d. Poverty:** What questions were used to assess poverty? Since you have said there were only 43 questions in your questionnaire, it appears that a single question about daily income was used to identify poverty. If this is true, a line of explanation to say that you are aware of the limitation of using a single question to determine poverty status will make it a bit more credible.
**Answer**: Many thanks. Yes, you are right. It was based on only one question, i.e., family income per person per day. Poverty was defined based on The World Bank definition, i.e., a family that earns <150 Afghanis (<2.15 USD) per person per day. Now this has been added in the “Limitation” section to make it a bit more credible.

**e. Table 2:** The label of the summary score at the bottom says 'Good score < 8.4' and 'Poor score >= 8.4' - should it not be the other way round?
**Answer**: Sorry for the confusion. Based on your previous comment, we have now revised it and it has been made clear as follows:

- Poor knowledge (score 0–7)
- Good knowledge (score 8–15)

**f.** Some questions in the tables are difficult to comprehend, and will probably benefit from better translations: for instance, 'Worrying is the impression of CL' or 'Worktime preference' or 'Sleeping disorders'. Please check all questions again to make things clearer for the readers.
**Answer**: Sorry for the misunderstanding. Now it has been translated properly as follows:

“Worrying is the impression of CL” is changed to “CL can cause anxiety”, “Sleeping disorders” is changed to “Sleeping outdoors”, “Worktime preference” is changed to “Working time”.

**g.** In general, responses to several questions are puzzling, and it is not clear that the authors have noticed them or understand why the responses are such. To point out just one instance, in Table 4, only 4 have said they have participated in CL control activities, but 182 have said yes to IRS in the past 12 months and 959 have said yes to use of bednets. Yet, there is no comment about this discrepancy.

**Answer**: Many thanks for raising the important point. Sorry for not clarifying it in the manuscript. Now it is clarified as follows: CL control activities are rarely implemented in these areas. Only 4 of our study participants said that they have participated in these activities. However, the IRS is applied by some local residents without any technical support from the experts. The local people in these study areas usually buy the insecticide from the market without a prescription and use it by themselves, mostly for the prevention of dangerous insects, such as scorpions and poisonous spiders.

We would like to make a more general observation deriving from such examples. The main value of such a paper for a global audience is to gain understanding of the nuances of the research findings, beyond just the numbers, about what is happening in Afghanistan, which is so inaccessible otherwise. Surely, the researchers have deeper insights into the local beliefs, customs, practices. It would be valuable to bring out some of this, such as in the Discussions section, and explain at least some of the relevant findings, using your own understanding. Without such explanations, readers could feel frustrated. One of the reviewers has also pointed out something similar.

**Answer**: Many thanks. Now the information has been added to the Discussion section.

**h.** In Table 5, one surprising statistically insignificant association reported for 'Literacy level' turns out to be a mistake in calculating the COR (the COR should be 3.5, not 1.1, it appears). This should be corrected, and reflected in results section. This is the strongest association in the entire bivariate/multivariate analysis, but has been missed. May be good to recheck all ORs.

**Answer**: Many thanks. Now all the data analyses have been revised in Tables 5, 6, and 7. The tables and descriptions have been thoroughly re-checked.

**i.** For some unexplained reason, the reference categories in COR seem to be different across tables 5, 6 and 7 for the same variables. For instance, see the reference categories for Literacy Level across the tables: Illiterate is '1' in some tables, and Literate is '1' in others. Either a correction or an explanation would be useful.

**Answer**: Many thanks. After thorough revision, now all the reference corrected have been corrected and uniform.

Overall, the study probably has significant methodological limitations which are not possible to correct during analysis. However, the paper is still valuable, considering that it is about a population which is so poorly understood and resourced. If the authors can pay a bit more attention to the mistakes and lack of clarity such as those mentioned above, it will enhance the value of the paper for the interested reader.

**Reviewers' comments:**

Reviewer's Responses to Questions

**Key Review Criteria Required for Acceptance?**

As you describe the new analyses required for acceptance, please consider the following:

**Methods:**

**Reviewer #1:** Significant revisions were made to the manuscript since the first revision and I do believe that it is stronger. I have a couple of questions that do not seem to have been addressed adequately.
**1)** Methods- Definitions- Previously I had inquired about how the definitions were determined as the text states that they are the mean values. If this were the case, roughly 50% would have been expected to score above (barring significant difference between mean and median, which is probably limited given the bounded nature of the result. Also, it is unclear why mean was chosen in the first place rather than median). The answer provided was just a reiteration that it was the mean value. It appears that the actual answer is that this is derived from external data and represents the mean value obtained in Ethiopia in reference #30 (Geto et al.) This requires explanation in the text about the source of the cut-off as it is currently implied that it is derived internally, and raises the question of why it was determined that the values determined in the population in Ethiopia would be valid in Afghanistan (They might be and if there is a reason it should be stated. I might imagine something like "to allow direct comparisons between the populations").

**Answer**: Many thanks for the useful comment, and sorry for not answering it well. Based on your comment, we have now completely revised the KAP cut-off values for the manuscript. We used the cut-off values for this study based on the number of questions asked and their answers in our research. We have now defined these cut-off values in the Materials and Methods sections of our manuscript as follows:

- For knowledge, there were 15 questions. Scores were 0–15. So, the maximum score was 15. Poor knowledge about CL was defined as a study participant who scored 0–7 points. Good knowledge about CL was defined as a study participant who scored 8–15 points.
- For attitude, there were 12 questions. Scores were 0–12. So, the maximum score was 12. Negative attitude about CL was defined as a study participant who scored 0–5 points. A positive attitude about CL was defined as a study participant who scored 6–12 points.
- For practice, there were 8 questions. Scores were 0–8. So, the maximum score was 8. Poor prevention practice towards CL was defined as a study participant who scored 0–3 points. Good prevention practice about CL was defined as a study participant who scored 4–8 points.

**Results:**

**Reviewer #1:** I did not see any revision to the rare statistical results that looked unusual that were mentioned in my prior review. I trust that they were verified.
I do notice a particularly unusual result that I have to question. In Table 2- it seems extremely unlikely that in a survey of 2118 individual that 0/2118 would agree that CL was transmitted by sandfly. It seems that even if they had no knowledge of the disease whatsoever, someone would have answered yes, even if by accident.

**Answer**: Many thanks for the important comment, and sorry for not making it clear. Now the question has been corrected in the table.

In fact, this question was open-ended, not multiple choice. Our question was “What vector transmits the CL?”. Most of them answered “I don’t know” while a few answered “mosquitoes”. Those people did not have any information regarding “sandfly” in the transmission of CL.

Hope all the questions are answered.
