# Supplementary material for: Knowledge, attitude, and prevention practices of cutaneous leishmaniasis in highly-endemic rural areas of Kandahar province, Afghanistan: A large cross-sectional community-based study
Source: PLoS Negl Trop Dis. 2026 Jun 3;20(6):e0013575. doi: 10.1371/journal.pntd.0013575 (PMC13245870; doi:10.1371/journal.pntd.0013575)
Supplement: Peer review history

# Peer review: Decision Letter 0

**DOI:** 10.1371/journal.pntd.0013575.r001

**Authors:** Abhay Satoskar

8 Dec 2025

PNTD-D-25-01655

Knowledge, attitude, and prevention practices of cutaneous leishmaniasis in highly-endemic rural areas of Kandahar province, Afghanistan: A large cross-sectional community-based study.

PLOS Neglected Tropical Diseases

Dear Dr. Rahimi,

Thank you for submitting your manuscript to PLOS Neglected Tropical Diseases. After careful consideration, we feel that it has merit but does not fully meet PLOS Neglected Tropical Diseases's publication criteria as it currently stands. Therefore, we invite you to submit a revised version of the manuscript that addresses the points raised during the review process.

Please submit your revised manuscript by Feb 06 2026 11:59PM. If you will need more time than this to complete your revisions, please reply to this message or contact the journal office at plosntds@plos.org. When you're ready to submit your revision, log on to https://www.editorialmanager.com/pntd/ and select the 'Submissions Needing Revision' folder to locate your manuscript file.

Please include the following items when submitting your revised manuscript:

* A rebuttal letter that responds to each point raised by the editor and reviewer(s). You should upload this letter as a separate file labeled 'Response to Reviewers'. This file does not need to include responses to any formatting updates and technical items listed in the 'Journal Requirements' section below.

* A marked-up copy of your manuscript that highlights changes made to the original version. You should upload this as a separate file labeled 'Revised Manuscript with Track Changes'.

* An unmarked version of your revised paper without tracked changes. You should upload this as a separate file labeled 'Manuscript'.

If you would like to make changes to your financial disclosure, competing interests statement, or data availability statement, please make these updates within the submission form at the time of resubmission. Guidelines for resubmitting your figure files are available below the reviewer comments at the end of this letter.

We look forward to receiving your revised manuscript.

Kind regards,

Abhay R Satoskar

Section Editor

PLOS Neglected Tropical Diseases

Abhay Satoskar

Section Editor

PLOS Neglected Tropical Diseases

Shaden Kamhawi

co-Editor-in-Chief

PLOS Neglected Tropical Diseases

orcid.org/0000-0003-4304-636XX

Paul Brindley

co-Editor-in-Chief

PLOS Neglected Tropical Diseases

orcid.org/0000-0003-1765-0002

**Journal Requirements:**

If the reviewer comments include a recommendation to cite specific previously published works, please review and evaluate these publications to determine whether they are relevant and should be cited. There is no requirement to cite these works unless the editor has indicated otherwise.

1) Please upload all main figures as separate Figure files in .tif or .eps format. For more information about how to convert and format your figure files please see our guidelines:

https://journals.plos.org/plosntds/s/figures

2) Some material included in your submission may be copyrighted. According to PLOSu2019s copyright policy, authors who use figures or other material (e.g., graphics, clipart, maps) from another author or copyright holder must demonstrate or obtain permission to publish this material under the Creative Commons Attribution 4.0 International (CC BY 4.0) License used by PLOS journals. Please closely review the details of PLOSu2019s copyright requirements here: PLOS Licenses and Copyright. If you need to request permissions from a copyright holder, you may use PLOS's Copyright Content Permission form.

Please respond directly to this email and provide any known details concerning your material's license terms and permissions required for reuse, even if you have not yet obtained copyright permissions or are unsure of your material's copyright compatibility. Once you have responded and addressed all other outstanding technical requirements, you may resubmit your manuscript within Editorial Manager.

Potential Copyright Issues:

i) Figure 1. Please (a) provide a direct link to the base layer of the map (i.e., the country or region border shape) and ensure this is also included in the figure legend; and (b) provide a link to the terms of use / license information for the base layer image or shapefile. We cannot publish proprietary or copyrighted maps (e.g. Google Maps, Mapquest) and the terms of use for your map base layer must be compatible with our CC BY 4.0 license.

Note: if you created the map in a software program like R or ArcGIS, please locate and indicate the source of the basemap shapefile onto which data has been plotted.

If your map was obtained from a copyrighted source please amend the figure so that the base map used is from an openly available source. Alternatively, please provide explicit written permission from the copyright holder granting you the right to publish the material under our CC BY 4.0 license.

If you are unsure whether you can use a map or not, please do reach out and we will be able to help you. The following websites are good examples of where you can source open access or public domain maps:

* U.S. Geological Survey (USGS) - All maps are in the public domain. (http://www.usgs.gov)

* PlaniGlobe - All maps are published under a Creative Commons license so please cite u201cPlaniGlobe, http://www.planiglobe.com, CC BY 2.0u201d in the image credit after the caption. (http://www.planiglobe.com/?lang=enl)

* Natural Earth - All maps are public domain. (http://www.naturalearthdata.com/about/terms-of-use/).

**Reviewers' comments:**

Reviewer's Responses to Questions

**Key Review Criteria Required for Acceptance?**

As you describe the new analyses required for acceptance, please consider the following:

**Methods**

-Are the objectives of the study clearly articulated with a clear testable hypothesis stated?

-Is the study design appropriate to address the stated objectives?

-Is the population clearly described and appropriate for the hypothesis being tested?

-Is the sample size sufficient to ensure adequate power to address the hypothesis being tested?

-Were correct statistical analysis used to support conclusions?

-Are there concerns about ethical or regulatory requirements being met?

Reviewer #1: The methods are generally clear. There is no testable hypothesis, though as a descriptive work, the design is appropriate.

I had a couple of questions about findings that might be related to methods, or results. I will mention them here.

1) Line 279- The overall rate of 14.8% of study participants had a score classified as "good knowledge" of CL. But in the methods section (line 184) the definition of "good knowledge" is defined as the mean value on the survey instrument. This would seem to imply that necessarily 50% should have had good knowledge. If the method for this determination, for example taking the mean from some other validation or population, was used, please clarify.

2) Statistical analysis- It is striking that so many confidence intervals include or barely exclude 1, but the corresponding p values are highly significant. It is possible that this is from rounding of log ORs. It would be helpful the authors could comment.

Reviewer #2: The objectives of the study are clearly outlined. However, for improved clarity, it would be helpful to include the specific hypothesis that was tested. The study design appears appropriate.

The section under "Study Population and Sample Size Calculation" should be reworded for enhanced clarity. Specifically, the inclusion and exclusion criteria need to be clearly stated. For instance, did the study exclude individuals aged '18 years or younger'? Was the study population drawn from all residents of the two districts, or only those living in the rural areas, as mentioned in line 152? If the latter, how were these ‘rural areas’ defined or selected?

Additionally, please specify the variables used in your sample size calculation. Was the final calculated sample size 2183?

Lastly, in line 158, if the authors intend to refer to ‘identification information,’ it would be clearer to explicitly use the term “identification information” rather than just “information.”

**********

**Results**

-Does the analysis presented match the analysis plan?

-Are the results clearly and completely presented?

-Are the figures (Tables, Images) of sufficient quality for clarity?

Reviewer #1: The results are clearly presented.

The discussion would benefit from restricting somewhat. Currently there are long stretches of reciting extensive results from other studies that may or may not have relevance here. I would suggest selecting which results the authors wish to present and framing them as comparisons or contrasts to the results found in this survey.

Reviewer #2: The results are presented clearly and concisely

**********

**Conclusions**

-Are the conclusions supported by the data presented?

-Are the limitations of analysis clearly described?

-Do the authors discuss how these data can be helpful to advance our understanding of the topic under study?

-Is public health relevance addressed?

Reviewer #1: (No Response)

Reviewer #2: In lines 357-359, the statement, “Based on the results of our study, the majority of the rural population of Daman and Arghandab districts of Kandahar province in Afghanistan had poor knowledge about CL, a negative attitude towards CL, and poor prevention practices towards CL,” should more clearly specify the study population. As noted in a previous comment, it is important to clarify whether this refers to the entire rural population of Daman and Arghandab districts or to the broader population of both districts.

Additionally, it would enhance the clarity of the conclusion if you briefly highlight, at the beginning of the section, the statistically significant factors associated with your findings. This would provide readers with a clearer summary of the key takeaways from your study.

**********

**Editorial and Data Presentation Modifications?**

Use this section for editorial suggestions as well as relatively minor modifications of existing data that would enhance clarity. If the only modifications needed are minor and/or editorial, you may wish to recommend “Minor Revision” or “Accept”.

Reviewer #1: (No Response)

Reviewer #2: The manuscript would benefit from language editing to improve clarity and readability in several sections.

**********

**Summary and General Comments**

Use this section to provide overall comments, discuss strengths/weaknesses of the study, novelty, significance, general execution and scholarship. You may also include additional comments for the author, including concerns about dual publication, research ethics, or publication ethics. If requesting major revision, please articulate the new experiments that are needed.

Reviewer #1: (No Response)

Reviewer #2: The authors have made a good effort in bridging a gap in available data related to leishmaniasis in a selected region in Afghanistan. Attending to the minor revisions mentioned will improve the manuscript.

**********

PLOS authors have the option to publish the peer review history of their article (what does this mean?). If published, this will include your full peer review and any attached files.

If you choose “no”, your identity will remain anonymous but your review may still be made public.

**Do you want your identity to be public for this peer review?** For information about this choice, including consent withdrawal, please see our Privacy Policy.

Reviewer #1: No

Reviewer #2: No

**Figure resubmission:**

While revising your submission, we strongly recommend that you use PLOS’s NAAS tool (https://ngplosjournals.pagemajik.ai/artanalysis) to test your figure files. NAAS can convert your figure files to the TIFF file type and meet basic requirements (such as print size, resolution), or provide you with a report on issues that do not meet our requirements and that NAAS cannot fix.

After uploading your figures to PLOS’s NAAS tool - https://ngplosjournals.pagemajik.ai/artanalysis, NAAS will process the files provided and display the results in the "Uploaded Files" section of the page as the processing is complete. If the uploaded figures meet our requirements (or NAAS is able to fix the files to meet our requirements), the figure will be marked as "fixed" above. If NAAS is unable to fix the files, a red "failed" label will appear above. When NAAS has confirmed that the figure files meet our requirements, please download the file via the download option, and include these NAAS processed figure files when submitting your revised manuscript.

**Reproducibility:**

To enhance the reproducibility of your results, we recommend that authors of applicable studies deposit laboratory protocols in protocols.io, where a protocol can be assigned its own identifier (DOI) such that it can be cited independently in the future. Additionally, PLOS ONE offers an option to publish peer-reviewed clinical study protocols. Read more information on sharing protocols at https://plos.org/protocols?utm_medium=editorial-email&utm_source=authorletters&utm_campaign=protocols

# Author comment: Author response to Decision Letter 1

**DOI:** 10.1371/journal.pntd.0013575.r002

29 Dec 2025

Attachment Submitted filename: Response to reviewers.docx

# Peer review: Decision Letter 1

**DOI:** 10.1371/journal.pntd.0013575.r003

**Authors:** Sridhar Srikantiah

15 Apr 2026

PNTD-D-25-01655R1

Knowledge, attitude, and prevention practices of cutaneous leishmaniasis in highly-endemic rural areas of Kandahar province, Afghanistan: A large cross-sectional community-based study.

PLOS Neglected Tropical Diseases

Dear Dr. Rahimi,

Thank you for submitting your manuscript to PLOS Neglected Tropical Diseases. After careful consideration, we feel that it has merit but does not fully meet PLOS Neglected Tropical Diseases's publication criteria as it currently stands. Therefore, we invite you to submit a revised version of the manuscript that addresses the points raised during the review process.

Please submit your revised manuscript by May 15 2026 11:59PM. If you will need more time than this to complete your revisions, please reply to this message or contact the journal office at plosntds@plos.org. When you're ready to submit your revision, log on to https://www.editorialmanager.com/pntd/ and select the 'Submissions Needing Revision' folder to locate your manuscript file.

Please include the following items when submitting your revised manuscript:

* A letter that responds to each point raised by the editor and reviewer(s). You should upload this letter as a separate file labeled 'Response to Reviewers'. This file does not need to include responses to any formatting updates and technical items listed in the 'Journal Requirements' section below.

* A marked-up copy of your manuscript that highlights changes made to the original version. You should upload this as a separate file labeled 'Revised Manuscript with Track Changes'.

* An unmarked version of your revised paper without tracked changes. You should upload this as a separate file labeled 'Manuscript'.

If you would like to make changes to your financial disclosure, competing interests statement, or data availability statement, please make these updates within the submission form at the time of resubmission. Guidelines for resubmitting your figure files are available below the reviewer comments at the end of this letter.

We look forward to receiving your revised manuscript.

Kind regards,

Sridhar Srikantiah, M.D.

Guest Editor

PLOS Neglected Tropical Diseases

Hira Nakhasi

Section Editor

PLOS Neglected Tropical Diseases

Shaden Kamhawi

co-Editor-in-Chief

PLOS Neglected Tropical Diseases

orcid.org/0000-0003-4304-636XX

Paul Brindley

co-Editor-in-Chief

PLOS Neglected Tropical Diseases

orcid.org/0000-0003-1765-0002

**Additional Editor Comments:**

We thank you for your efforts to respond to the reviewers' comments with reference to your submitted manuscript.

We request your further attention to the following:

a.Selection of cut-offs for KAP scores: It appears that you have probably used a questionnaire in your study which is identical to that used in an Ethiopian study published in 2024 (Geto et al). You also state in your response to one of the reviewers that you used the same cut-offs for separating good and poor KAP scores as were used in the Geto et al paper. At the same time, in the Definitions section, you state that the cut-offs (8.4, 30.86, and 5.0) were the mean values of the respective K, A, P scores. By default it will be interpreted that these are mean scores from your study. It is virtually impossible for mean values from two different studies to be the same, even if they used identical tools.

a1. Could you confirm that you have used the cut-offs from the Geto et al paper, and that the cut-offs are not the mean scores from your study? If this is true, please state clearly in the Definitions sections that the cut-offs used are the same as in the in the Geto et al study, and provide the reason/justification for using the same cut-offs. For instance, you can say that you used the same cut-offs to make comparisons easier.

a2. If the cut-offs are not the means of the scores from your study data, and since the main purpose of the paper is to provide descriptive analysis, it would be appropriate to provide the basic descriptive figures for at least the summary K, A, P scores - mean, median, mode, in the respective tables or in the Results section. This will help the reader understand the distribution of response scores more clearly.

b. The Attitude questions are based on Likert scales. It has not been explained how the responses were scored using the Likert scales:

b1. Which questions scored high for 'strongly agree' or for 'strongly disagree', for instance?

b2. What was the maximum possible score?

c. Table 1: percentages are either wrongly calculated or labelled: The percentages are correctly calculated for the column 'Total', but are differently calculated for the columns 'Daman' and 'Arghandab': here, each cell carries row percentages, but the column label indicates that they are column percentages. In this situation, column percentages will make more useful sense than row percentages. The column labels are fine, just change the cell percentages to row percentages for the two district specific columns. This will make it easier for the reader to judge the differences between the two districts.

d. Poverty: What questions were used to assess poverty? Since you have said there were only 43 questions in your questionnaire, it appears that a single question about daily income was used to identify poverty. If this is true, a line of explanation to say that you are aware of the limitation of using a single question to determine poverty status will make it a bit more credible.

e. Table 2: The label of the summary score at the bottom says 'Good score < 8.4' and 'Poor score >= 8.4' - should it not be the other way round?

f. Some questions in the tables are difficult to comprehend, and will probably benefit from better translations: for instance, 'Worrying is the impression of CL' or 'Worktime preference' or 'Sleeping disorders'. Please check all questions again to make things clearer for the readers.

g. In general, responses to several questions are puzzling, and it is not clear that the authors have noticed them or understand why the responses are such. To point out just one instance, in Table 4, only 4 have said they have participated in CL control activities, but 182 have said yes to IRS in the past 12 months and 959 have said yes to use of bednets. Yet, there is no comment about this discrepancy.

We would like to make a more general observation deriving from such examples. The main value of such a paper for a global audience is to gain understanding of the nuances of the research findings, beyond just the numbers, about what is happening in Afghanistan, which is so inaccessible otherwise. Surely, the researchers have deeper insights into the local beliefs, customs, practices. It would be valuable to bring out some of this, such as in the Discussions section, and explain at least some of the relevant findings, using your own understanding. Without such explanations, readers could feel frustrated. One of the reviewers has also pointed out something similar.

h. In Table 5, one surprising statistically insigificant association reported for 'Literacy level' turns out to be a mistake in calculating the COR (the COR should be 3.5, not 1.1, it appears). This should be corrected, and reflected in results section. This is the strongest association in the entire bivariate/mutivariate analysis, but has been missed. May be good to recheck all ORs.

i. For some unexplained reason, the reference categories in COR seem to be different across tables 5, 6 and 7 for the same variables. For instance, see the reference categories for Literacy Level across the tables: Illiterate is '1' in some tables, and Literate is '1' in others. Either a correction or an explantion would be useful.

Overall, the study probably has significant methodological limitations which are not possible to correct during analysis. However, the paper is still valuable, considering that it is about a population which is so poorly understood and resourced. If the authors can pay a bit more attention to the mistakes and lack of clarity such as those mentioned above, it will enhance the value of the paper for the interested reader.

Thank you again, for your effort and attention.

**Reviewers' comments:**

Reviewer's Responses to Questions

**Key Review Criteria Required for Acceptance?**

As you describe the new analyses required for acceptance, please consider the following:

**Methods:**

-Are the objectives of the study clearly articulated with a clear testable hypothesis stated?

-Is the study design appropriate to address the stated objectives?

-Is the population clearly described and appropriate for the hypothesis being tested?

-Is the sample size sufficient to ensure adequate power to address the hypothesis being tested?

-Were correct statistical analysis used to support conclusions?

-Are there concerns about ethical or regulatory requirements being met?

Reviewer #1: Significant revisions were made to the manuscript since the first revision and I do believe that it is stronger. I have a couple of questions that do not seem to have been addressed adequately.

1) Methods- Definitions- Previously I had inquired about how the definitions were determined as the text states that they are the mean values. If this were the case, roughly 50% would have been expected to score above (barring significant difference between mean and median, which is probably limited given the bounded nature of the result. Also, it is unclear why mean was chosen in the first place rather than median). The answer provided was just a reiteration that it was the mean value. It appears that the actual answer is that this is derived from external data and represents the mean value obtained in Ethiopia in reference #30 (Geto et al.) This requires explanation in the text abut the source of the cut-off as it is currently implied that it is derived internally, and raises the question of why it was determined that the values determined in the population in Ethiopia would be valid in Afghanistan (They might be and if there is a reason it should be stated. I might imagine something like "to allow direct comparisons between the populations").

**********

**Results:**

-Does the analysis presented match the analysis plan?

-Are the results clearly and completely presented?

-Are the figures (Tables, Images) of sufficient quality for clarity?

Reviewer #1: I did not see any revision to the rare statistical results that looked unusual that were mentioned in my prior review. I trust that they were verified.

I do notice a particularly unusual result that I have to question. In Table 2- it seems extremely unlikely that in a survey of 2118 individual that 0/2118 would agree that CL was transmitted by sandfly. It seems that even if the had no knowledge of the disease whatsoever, someone would have answered yes, even if by accident.

**********

**Conclusions:**

-Are the conclusions supported by the data presented?

-Are the limitations of analysis clearly described?

-Do the authors discuss how these data can be helpful to advance our understanding of the topic under study?

-Is public health relevance addressed?

Reviewer #1: (No Response)

**********

**Editorial and Data Presentation Modifications?**

Use this section for editorial suggestions as well as relatively minor modifications of existing data that would enhance clarity. If the only modifications needed are minor and/or editorial, you may wish to recommend “Minor Revision” or “Accept”.

Reviewer #1: (No Response)

**********

**Summary and General Comments:**

Use this section to provide overall comments, discuss strengths/weaknesses of the study, novelty, significance, general execution and scholarship. You may also include additional comments for the author, including concerns about dual publication, research ethics, or publication ethics. If requesting major revision, please articulate the new experiments that are needed.

Reviewer #1: (No Response)

**********

PLOS authors have the option to publish the peer review history of their article (what does this mean?). If published, this will include your full peer review and any attached files.

If you choose “no”, your identity will remain anonymous but your review may still be made public.

**Do you want your identity to be public for this peer review?** For information about this choice, including consent withdrawal, please see our Privacy Policy.

Reviewer #1: No

[NOTE: If reviewer comments were submitted as an attachment file, they will be attached to this email and accessible via the submission site. Please log into your account, locate the manuscript record, and check for the action link "View Attachments". If this link does not appear, there are no attachment files.]

**Figure resubmission:**

While revising your submission, we strongly recommend that you use PLOS’s NAAS tool (https://ngplosjournals.pagemajik.ai/artanalysis) to test your figure files. NAAS can convert your figure files to the TIFF file type and meet basic requirements (such as print size, resolution), or provide you with a report on issues that do not meet our requirements and that NAAS cannot fix.

After uploading your figures to PLOS’s NAAS tool - https://ngplosjournals.pagemajik.ai/artanalysis, NAAS will process the files provided and display the results in the "Uploaded Files" section of the page as the processing is complete. If the uploaded figures meet our requirements (or NAAS is able to fix the files to meet our requirements), the figure will be marked as "fixed" above. If NAAS is unable to fix the files, a red "failed" label will appear above. When NAAS has confirmed that the figure files meet our requirements, please download the file via the download option, and include these NAAS processed figure files when submitting your revised manuscript.

**Reproducibility:**

To enhance the reproducibility of your results, we recommend that authors of applicable studies deposit laboratory protocols in protocols.io, where a protocol can be assigned its own identifier (DOI) such that it can be cited independently in the future. Additionally, PLOS ONE offers an option to publish peer-reviewed clinical study protocols. Read more information on sharing protocols at https://plos.org/protocols?utm_medium=editorial-email&utm_source=authorletters&utm_campaign=protocols

# Author comment: Author response to Decision Letter 2

**DOI:** 10.1371/journal.pntd.0013575.r004

10 May 2026

Attachment Submitted filename: Response_to_reviewers_auresp_2.docx

# Editor report: Decision Letter 2

**DOI:** 10.1371/journal.pntd.0013575.r005

**Authors:** Sridhar Srikantiah

20 May 2026

Dear Professor Rahimi,

We are pleased to inform you that your manuscript 'Knowledge, attitude, and prevention practices of cutaneous leishmaniasis in highly-endemic rural areas of Kandahar province, Afghanistan: A large cross-sectional community-based study.' has been provisionally accepted for publication in PLOS Neglected Tropical Diseases.

Before your manuscript can be formally accepted you will need to complete some formatting changes, which you will receive in a follow up email. A member of our team will be in touch with a set of requests.

Please note that your manuscript will not be scheduled for publication until you have made the required changes, so a swift response is appreciated.

IMPORTANT: The editorial review process is now complete. PLOS will only permit corrections to spelling, formatting or significant scientific errors from this point onwards. Requests for major changes, or any which affect the scientific understanding of your work, will cause delays to the publication date of your manuscript.

Should you, your institution's press office or the journal office choose to press release your paper, you will automatically be opted out of early publication. We ask that you notify us now if you or your institution is planning to press release the article. All press must be co-ordinated with PLOS.

Thank you again for supporting Open Access publishing; we are looking forward to publishing your work in PLOS Neglected Tropical Diseases.

Best regards,

Sridhar Srikantiah, M.D.

Guest Editor

PLOS Neglected Tropical Diseases

Hira Nakhasi

Section Editor

PLOS Neglected Tropical Diseases

Shaden Kamhawi

co-Editor-in-Chief

PLOS Neglected Tropical Diseases

orcid.org/0000-0003-4304-636XX

Paul Brindley

co-Editor-in-Chief

PLOS Neglected Tropical Diseases

orcid.org/0000-0003-1765-0002

***********************************************************

p.p1 {margin: 0.0px 0.0px 0.0px 0.0px; line-height: 16.0px; font: 14.0px Arial; color: #323333; -webkit-text-stroke: #323333}span.s1 {font-kerning: none

# Editor report: Acceptance letter

**DOI:** 10.1371/journal.pntd.0013575.r006

**Authors:** Sridhar Srikantiah

Dear Professor Rahimi,

We are delighted to inform you that your manuscript, "Knowledge, attitude, and prevention practices of cutaneous leishmaniasis in highly-endemic rural areas of Kandahar province, Afghanistan: A large cross-sectional community-based study.," has been formally accepted for publication in PLOS Neglected Tropical Diseases.

We have now passed your article onto the PLOS Production Department who will complete the rest of the publication process. All authors will receive a confirmation email upon publication.

The corresponding author will soon be receiving a typeset proof for review, to ensure errors have not been introduced during production. Please review the PDF proof of your manuscript carefully, as this is the last chance to correct any scientific or type-setting errors. Please note that major changes, or those which affect the scientific understanding of the work, will likely cause delays to the publication date of your manuscript. Note: Proofs for Front Matter articles (Editorial, Viewpoint, Symposium, Review, etc...) are generated on a different schedule and may not be made available as quickly.

Soon after your final files are uploaded, the early version of your manuscript will be published online unless you opted out of this process. The date of the early version will be your article's publication date. The final article will be published to the same URL, and all versions of the paper will be accessible to readers.

For Research Articles, you will receive an invoice from PLOS for your publication fee after your manuscript has reached the completed accept phase. If you receive an email requesting payment before acceptance or for any other service, this may be a phishing scheme. Learn how to identify phishing emails and protect your accounts at https://explore.plos.org/phishing.

Thank you again for supporting open-access publishing; we are looking forward to publishing your work in PLOS Neglected Tropical Diseases.

Best regards,

Shaden Kamhawi

co-Editor-in-Chief

PLOS Neglected Tropical Diseases

Paul Brindley

co-Editor-in-Chief

PLOS Neglected Tropical Diseases
